# Rotenone-induced oxidative stress triggers peroxisomal alterations in BV-2 microglial cells

**DOI:** 10.3389/fncel.2026.1941513

**Published:** 2026-08-21

**Authors:** Alessio Valenza, Maurizio Muzzi, Francesca Rendina, Irene Montali, Daniele Pensabene, Anna Fracassi, Marco Segatto, Sandra Moreno

**Affiliations:** 1 Department of Science, University Roma Tre, Rome, Italy; 2 LIFE Institute for Research and Health Care—Santa Lucia IRCCS, Rome, Italy; 3 Department of Biosciences and Territory, University of Molise, Pesche, Italy; 4 Department of Neurology, Mitchell Center for Neurodegenerative Diseases, University of Texas Medical Branch, UTMB, Galveston, TX, United States; 5 Moody Brain Health Institute, UTMB, Galveston, TX, United States; 6 Graduate School of Biomedical Sciences, UTMB, Galveston, TX, United States

**Keywords:** microglia, mitochondria, oxidative stress, peroxisomes, rotenone

## Abstract

**Introduction:**

Peroxisomes are highly dynamic organelles that contribute to cellular homeostasis by coordinating lipid metabolism, reactive oxygen species handling, and adaptive responses to metabolic stress. Their plasticity is particularly relevant in the nervous system, where peroxisomes cooperate with mitochondria to maintain redox and metabolic balance in neuronal and glial cells. Peroxisome proliferator-activated receptor alpha (PPARα), together with its coactivator PGC-1α, represents a major transcriptional regulator of peroxisomal and mitochondrial metabolic programs.

**Methods:**

BV2 microglial cells were exposed to rotenone to investigate how mitochondrial oxidative stress influences the peroxisomal compartment. Mitochondrial and peroxisomal morphology, intracellular distribution, oxidative damage, and the expression of proteins involved in peroxisomal metabolism and PPARα signaling were evaluated.

**Results:**

Rotenone induced a stress-associated microglial phenotype characterized by cytoskeletal remodeling, mitochondrial network disruption, and oxidative damage. These alterations were accompanied by a coordinated reorganization of the peroxisomal compartment, including increased abundance of peroxisomal membrane markers, redistribution of peroxisomes toward the perinuclear region, and enhanced expression of enzymes associated with peroxisomal fatty acid oxidation. An increased spatial association between peroxisomes and mitochondria was also observed, suggesting that peroxisomal remodeling forms part of a broader inter-organelle adaptation to mitochondrial dysfunction. These changes occurred together with increased nuclear phosphorylated PPARα and enhanced PGC-1α immunoreactivity, supporting the engagement of a PPARα–PGC-1α-related transcriptional response.

**Discussion:**

Overall, our findings suggest that mitochondrial dysfunction in microglial cells activates a PPARα-associated adaptive program that promotes peroxisomal remodeling and strengthens mitochondria–peroxisome coordination. This response may represent an attempt to preserve lipid and redox homeostasis during cellular stress and identifies PPARα-regulated peroxisomal pathways as potentially relevant components of microglial metabolic adaptation in neurodegenerative conditions.

## 1 Introduction

Peroxisomes are single-membrane-bound organelles (0.1–1 μm in diameter), ubiquitous in eukaryotic cells, that are essential for several catabolic and anabolic pathways, including lipid and reactive oxygen species (ROS) metabolism. Indeed, they participate in α- and β-oxidation of fatty acids and in the biosynthesis of ether phospholipid (plasmalogens) and docosahexaenoic acid, while acting as key regulators of cellular redox homeostasis, primarily through catalase activity (Schrader et al., 2015). Their biogenesis proceeds through two principal pathways: growth and division of pre-existing peroxisomes, and *de novo* formation from the endoplasmic reticulum, with additional contribution by mitochondria-derived vesicles (Kumar et al., 2024). A distinctive feature of peroxisomes is the post-translational import of fully folded membrane and matrix proteins. Importantly, the peroxisomal matrix includes specialized enzymatic machinery dedicated to hydrogen peroxide metabolism, with both H_2_O_2_-generating and -scavenging enzymes (Deb et al., 2021).

These organelles are remarkably dynamic, as they respond to environmental and intracellular cues by rapidly modulating their abundance, morphology, and functional properties, thus adapting to cellular demands (Carmichael and Schrader, 2022). Beyond changes in size and number, peroxisomes also undergo intracellular redistribution, involving microtubule-dependent long-range trafficking (Schrader et al., 1996). In neurons, under basal conditions, peroxisomes are predominantly localized within the soma, where their proximity to the plasma membrane may facilitate efficient processing and turnover of membrane-associated lipids (Wang et al., 2018). However, peroxisomal spatial organization is cell-type dependent, and emerging evidence suggests that its distribution and dynamics may also vary in glial populations (Rhoads et al., 2025). Notwithstanding their specific biochemical functions, they also operate within an integrated inter-organellar communication network, essential for cellular homeostasis. Peroxisomes interact with the endoplasmic reticulum, mitochondria, lysosomes, and lipid droplets, to coordinate lipid metabolism and redox balance (Schrader et al., 2020; Wanders et al., 2023). This crosstalk involves membrane contact sites, specialized regions where opposing organelle membranes are approximately 10–30 nm apart and interact through tethering proteins or specific membrane lipids (Scorrano et al., 2019).

Peroxisomes contribute to metabolic processes crucial to the brain function, supporting both neuronal and glial cells, i.e., contributing to the biosynthesis of plasmalogens, important components of myelin membranes. Given its high lipid content, the brain tissue is especially vulnerable to oxidative damage (Kumar et al., 2024), and alterations in peroxisomal lipid metabolism may even increase such suisceptibility. Consistent with their functional relevance, peroxisomal dysfunction associated with congenital disorders results in severe neurological abnormalities (Wanders et al., 2018; Honsho et al., 2020). Moreover, major neurodegenerative disorders, including Alzheimer’s disease (AD), Parkinson’s disease (PD), and Multiple Sclerosis (MS) are characterized by peroxisomal alterations (Zalckvar and Schuldiner, 2022). Notably, accumulating evidence on experimental stress models indicates that peroxisome proliferation is part of an adaptive response aimed at reinforcing antioxidant capacity. Specifically, following oxidative insult, upregulation of peroxisome abundance correlates with enhanced resilience to ROS-mediated damage, whereas impaired peroxisomal dynamics increases cellular vulnerability (Delmaghani et al., 2015). Consistently, in our previous work using a transgenic mouse model of familial AD, we identified hippocampal peroxisomal alterations indicative of an adaptive remodeling in response to Aβ- and ROS-driven mitochondrial stress, further supporting a role for peroxisomes in age-related disorders (Fanelli et al., 2013).

Importantly, peroxisomes are involved in immune and inflammatory signaling pathways relevant to neurodegenerative pathophysiology (Zarrouk et al., 2020). Accordingly, pharmacological activation of peroxisome proliferator-activated receptors (PPARs), which regulate peroxisomal metabolism and antioxidant responses, has been shown to exert neuroprotective effects in *in vivo* models of AD and PD (Katsouri et al., 2012; D’Orio et al., 2018). Noteworthy, PPARs and their coactivator 1-alpha (PGC-1α) are engaged not only in the expression of genes encoding peroxisomal proteins, but even in the modulation of mitochondrial biogenesis and function. Such action is crucial to the regulation of cellular energy homeostasis, particularly antioxidant defense, supporting the close interplay between peroxisomes and mitochondria (Valle et al., 2005; Fransen et al., 2017).

Mitochondrial dysfunction is a well-established driver of neuroinflammation. Alterations in mitochondrial biogenesis, fission–fusion dynamics, and electron transport chain (ETC) activity can initiate or amplify inflammatory responses within the nervous tissue (Chu, 2022). In this context, mitochondrial damage leads to elevated ROS generation, which activates glial cells, particularly microglia, thereby sustaining inflammatory signaling and contributing to neuronal dysfunction (Li et al., 2022). Several neurotoxins are commonly used to model mitochondrial degeneration *in vitro*, including paraquat, 1-methyl-4-phenylpyridinium (MPP^+^), 6-hydroxydopamine (6-OHDA), and rotenone (Rot) (Chithra et al., 2023). The latter, a naturally occurring isoflavone, derived from Fabaceae plants, is widely employed in PD models, due to its ability to inhibit mitochondrial Complex I of ETC, thereby impairing oxidative phosphorylation and inducing dopaminergic neurotoxicity (Li et al., 2003; Radad et al., 2019).

Despite evidence linking peroxisomal dysfunction to neurodegenerative disorders, how mitochondrial impairment affects peroxisomal organization and remodeling microglia during neuroinflammatory responses remains poorly understood. Given the central role of mitochondrial dysfunction and oxidative stress in Parkinson’s disease, we investigated how Rot-induced mitochondrial impairment alters the peroxisomal compartment in BV2 microglial cells. Specifically, we assessed changes in peroxisome abundance, enzymatic content, and intracellular distribution, including those potentially associated with Rot-induced microtubule destabilization, and examined their relationship with oxidative stress-driven microglial activation.

## 2 Materials and methods

### 2.1 Cell cultures and treatments

The BV2 microglial cell line present in this study was obtained from Interlab Cell Line Collection (ICLC, Genoa, Italy; ATL03001). Cells were cultured at 37 °C in a humidified atmosphere with 5% CO_2_ in high-glucose DMEM (D6429, Merck Life Science) supplemented with 10% (v/v) heat-inactivated fetal bovine serum (FBS) (F7524, Merck Life Science) and 1% (v/v) penicillin/streptomycin (P06-07100, PAN Biotech). For all experiments, cells were grown to 80–90% confluency and subjected to no more than 20 cell passages.

To assess rotenone-induced toxicity (R8875, Merck Life Science), cells were exposed to three concentrations (50 nM, 0.1 μM, 0.5 μM) for 24 h. 50 nM was selected for all subsequent analyzes. Experimental treatments (DMSO and Rotenone) were conducted in DMEM containing 2% FBS, and cells were analyzed after 24 h of incubation.

### 2.2 Cell count

BV2 cells were seeded in six-well plates, and morphological changes were monitored using a Primovert phase-contrast inverted microscope (Carl Zeiss) at 20× magnification. Representative images were captured for each experimental condition. For cell counting, cultures were incubated with trypsin for 5 min; the resulting cell suspensions were collected and homogenized. Cell numbers were determined using a Bürker chamber, performing three independent counts per experimental group, and the mean value was used for quantitative analysis.

### 2.3 Immunofluorescence and confocal microscopy

Immunofluorescence analyzes were carried out according to previously described protocols, with modifications (Pallottini et al., 2020; Colardo et al., 2023, 2025). Specifically, 150,000 cells were seeded onto sterilized poly-L-lysine–coated coverslips (P6282, Merck Life Science). After treatments, cells were fixed with 4% paraformaldehyde (D1408, Merck Life Science) in PBS for 5–10 min at room temperature (RT), followed by permeabilization with 0.1% Triton X-100 (X100, Merck Life Science) in PBS for 5 min. To minimize non-specific antibody binding, samples were incubated for 1 h at RT in a blocking solution of 3% bovine serum albumin (BSA) (A3912, Merck Life Science) and 0.1% Triton X-100 in PBS.

Cells were then incubated overnight at 4 °C with the primary antibodies listed in Table 1. After PBS washes, coverslips were incubated for 1 h at RT with appropriate fluorophore-conjugated secondary antibodies (goat anti-mouse Alexa Fluor 555, A28180; goat anti-rabbit Alexa Fluor 488, A27034; Thermo Fisher Scientific). Nuclei were counterstained with DAPI (D9542, Merck Life Science) and coverslips were mounted using Fluoroshield Mounting Medium (F6182, Merck Life Science). Fluorescence images were acquired using a confocal microscope (Eclipse Ti2, Nikon) equipped with 40× or 100× objectives. Image capturing was performed with NIS-Elements software (Nikon) on a Windows 10 platform, maintaining identical acquisition parameters across groups. Fluorescence intensity quantification and all morphometric analyzes were carried out using ImageJ (version 1.54d, NIH). For quantitative immunofluorescence analysis, regions of interest (ROIs) corresponding to individual cells were manually delineated based on cellular morphology. For each ROI, the mean fluorescence intensity (MFI) was calculated by dividing the integrated fluorescence intensity by the ROI area. Values were then normalized to the Ctrl group. Such approach provides a measure of the average fluorescence per unit area, minimizing the influence of differences in cell size and allowing comparison of marker expression levels between experimental groups.

**Table 1:** List of antibodies employed in this work.

Antibody	Application	Provider
4-HNE	IF 1:100	MA527570, Thermo Fisher Scientific
8-OH(d)G	IF 1:100	sc-66036, Santa Cruz Biotechnology
ACAA1	IF 1:200	ab154091, Abcam
ACOX1	IF 1:250	ab184032, Abcam
α-tubulin	IF 1:500	T5168, Sigma-Aldrich
Catalase	IF 1:500 WB 1:10.000	200401051, Rockland Immunochemicals
GAPDH	WB 1:10.000	ab8245, Abcam
IBA1	IF 1:50	sc-32725, Santa Cruz Biotechnology
PEX14	IF 1:200	ab183885, Abcam
PGC1α	IF 1:100	ab54481, Abcam
PMP70	IF 1:200	P0497, Merck
PPARα (pS12)	IF 1:100	ab3484, Abcam
TOMM20	IF 1:1000	ab209606, Abcam

### 2.4 MitoTracker™ Red CMXRos dye

MitoTracker probe was used to visualize the mitochondrial network. Briefly, cells were incubated with MitoTracker™ Red CMXRos (M7512, Thermo Fisher Scientific) according to the manufacturer’s instructions. After staining, cells were fixed, counterstained with DAPI, and analyzed by confocal microscopy. Evaluation of mitochondrial parameters was performed using the Mitochondrial Network Analysis tool (MiNA), a macro plugin based on the FIJI distribution of the ImageJ platform. Quantified parameters included Mitochondrial Count, Mean Aspect Ratio, Mean Form Factor, Number of Branches and Mean Branch Length. Mitochondrial Count indicates the number of individual mitochondrial objects detected in the analyzed region and provides an estimate of mitochondrial abundance and fragmentation. Mean Aspect Ratio describes mitochondrial elongation, with higher values corresponding to more elongated structures and lower values indicating rounder or more fragmented mitochondria. Mean Form Factor reflects mitochondrial shape complexity, considering both area and perimeter, and increases in more elongated or branched structures. The Number of Branches represents the total branches detected in the skeletonized network and is used as an indicator of mitochondrial interconnectivity, while Mean Branch Length indicates the average extension of these branches, providing information on the organization and continuity of the mitochondrial network (Koopman et al., 2005; Rumbeiha et al., 2023).

### 2.5 Western blotting

Cell lysis and Western blot analyzes were carried out with minor modifications to previously published protocols (Pallottini et al., 2020; Colardo et al., 2023, 2025). BV2 cells were disrupted by sonication for 30 s in CelLytic buffer (C2978, Sigma-Aldrich) supplemented with protease and phosphatase inhibitor cocktails, in order to obtain total protein extracts. Protein concentration was assessed using the Lowry method. Samples were subsequently combined with Laemmli buffer and denatured by heating at 95 °C for 5 min. Equal protein amounts (20 μg) were resolved on 10% SDS–PAGE gels and then transferred onto nitrocellulose membranes using the Trans-Blot Turbo system (Bio-Rad Laboratories). Membranes were blocked for 1 h at room temperature in 5% non-fat dry milk prepared in PBS containing 0.1% Tween-20 (PBS-T). They were then incubated overnight at 4 °C with the primary antibodies listed in Table 1, diluted in blocking solution. Following washes with PBS-T, membranes were incubated for 1 h at room temperature with HRP-conjugated secondary antibodies, namely anti-mouse (1706516, Bio-Rad Laboratories) or anti-rabbit (1706515, Bio-Rad Laboratories). Immunoreactive bands were visualized using Clarity ECL Western Blotting Substrate (1705061, Bio-Rad Laboratories). Images were acquired and densitometric analyzes were performed with ImageJ software (version 1.54d, National Institutes of Health). Densitometric data were reported as arbitrary units (a.u.), calculated as the ratio between the intensity of each target protein band and GAPDH, and normalized to Ctrl values.

### 2.6 Catalase activity assay

After treatments, differentiated BV2 cells were collected and centrifuged to obtain cell pellets. Pellets were processed for the determination of catalase enzymatic activity using a commercial Catalase Activity Assay Kit (MAK531, Merck Life Science), according to the manufacturer’s instructions.

### 2.7 Oil Red O staining

An Oil Red O (MAK560, Sigma-Aldrich) working solution was prepared by mixing 6 parts of stock solution with 4 parts of distilled water. The solution was allowed to stand for 10 min and filtered through Whatman No. 1 paper 15 min before use. Cells fixed in 4% paraformaldehyde were pre-treated with 60% isopropanol for 5 min, followed by incubation with the Oil Red O working solution for 15 min under gentle agitation. After staining, cells were washed five times with distilled water. Nuclei were counterstained with DAPI (1:1000). Coverslips were mounted using Fluoroshield and imaged at 100× magnification using the Eclipse Ti2 confocal microscope.

### 2.8 Scanning electron microscopy (SEM)

For ultrastructural analysis, cells grown on coverslips were fixed in 2.5% glutaraldehyde in 0.1 M cacodylate buffer (pH 7.4) for 45 min at 4 °C. Post-fixation was performed with 1% osmium tetroxide (OsO4) in the same buffer for 45 min at 4 °C in the dark. Samples were dehydrated through a graded ethanol series followed by immersion in hexamethyldisilazane. After air-drying, slides were mounted on metal stubs using double-sided conductive adhesive carbon discs and sputter-coated with gold (Emitech K550 Sputter Coater). Electron micrographs were acquired with a Helios 5 CX FIB/SEM, (Thermo Fisher Scientific) using secondary-electron detection at 5 kV.

### 2.9 Statistical analysis

Data are presented as mean ± standard deviation (SD). All experiments were performed on at least three independent biological replicates, unless otherwise specified in the figure legends. Each biological replicate corresponded to an independent cell culture experiment performed on different days. The exact number of replicates and/or analyzed cells is reported in the corresponding figure legends. Data distribution was assessed using the Shapiro–Wilk normality test. For comparisons between two experimental groups, an unpaired two-tailed Student’s *t*-test was used. When more than two experimental groups were compared, one-way analysis of variance (ANOVA) followed by Tukey’s *post hoc* test was applied. Statistical analyzes were performed using GraphPad Prism version 8.4.2 for Windows 10 (GraphPad Software, San Diego, CA, USA). A *p*-value < 0.05 was considered statistically significant.

## 3 Results

### 3.1 Rotenone treatment affects BV2 cell number and morphology, while inducing reactive microglial phenotype

**Figure 1:**
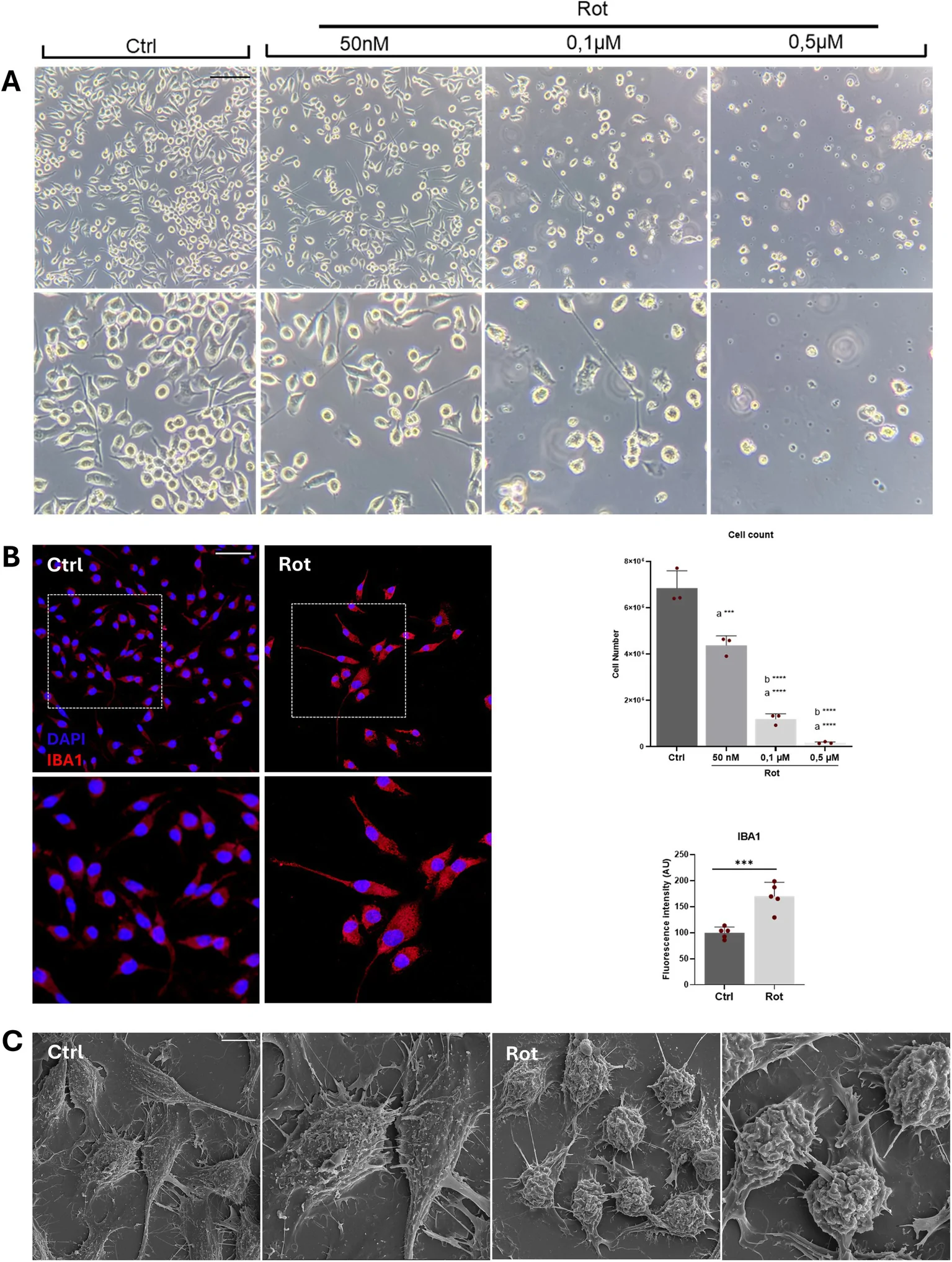
Rotenone reduces cell number in a dose-dependent manner and induces morphological alterations at sub-toxic concentration. **(A)** Representative brightfield images of a total cell count of BV2 cells treated with vehicle (Ctrl, DMSO) or Rot (50 nM, 0.1 μM, or 0.5 μM) for 24 h. Lower panels show higher-magnification views of the corresponding conditions. *N* = 3. Magnification: 20×. Scale bar = 50 μm. **(B)** Representative immunofluorescence images of IBA1-positive BV2 cells, with DAPI nuclear counterstaining, and quantitative analysis fluorescence intensity. *N* = 5. Magnification: 40×. Scale bar = 50 μm. **(C)** Representative SEM images showing ultrastructural changes in BV2 cells following rotenone treatment compared with control cells. Scale bar = 10 μm. Data are presented as mean ± SD. Dots represent individual biological replicates. Statistical analysis was performed using one-way ANOVA followed by Tukey’s *post hoc* test or unpaired Student’s *t*-test, as appropriate. ^*^p < 0.05; ^***^p < 0.001; ^****^p < 0.0001.

To determine an experimental condition capable of inducing inflammation-related changes without notable cytotoxicity, we evaluated the effects of graded rotenone concentrations (50 nM, 0.1 μM, and 0.5 μM) on BV2 total cell number. As shown by phase-contrast imaging (Figure 1A), rotenone induced a dose-dependent reduction in cell number compared to the control condition. While 0.1 μM and 0.5 μM caused a marked loss of cells, treatment with 50 nM rotenone resulted in a significant decrease in cell number without massive cell death. Consistently, morphological alterations became more dramatic with increasing rotenone concentrations. At 50 nM, BV2 cells exhibited activation-associated morphology, while largely preserving cell integrity. Indeed, higher magnification showed rounded morphology and reduction of cell processes and cell–cell contacts. In contrast, higher doses of rotenone led to pronounced shrinkage accompanied by increased cellular debris and detachment, consistent with cytotoxic damage. Based on these results, 50 nM rotenone was selected as the optimal concentration for subsequent experiments.

We investigated whether 50 nM Rot was sufficient to trigger microglial activation, by analyzing the expression of ionized calcium-binding adaptor molecule 1 (Iba1), a widely used marker involved in cytoskeletal remodeling, membrane ruffling and dynamic morphological changes, associated with activated microglia (Gheorghe et al., 2020). Immunofluorescence analysis (Figure 1B) revealed a significant increase in Iba1 fluorescence intensity in Rot-treated cells, compared to control, indicating a shift toward a reactive microglial phenotype.

**Figure 2:**
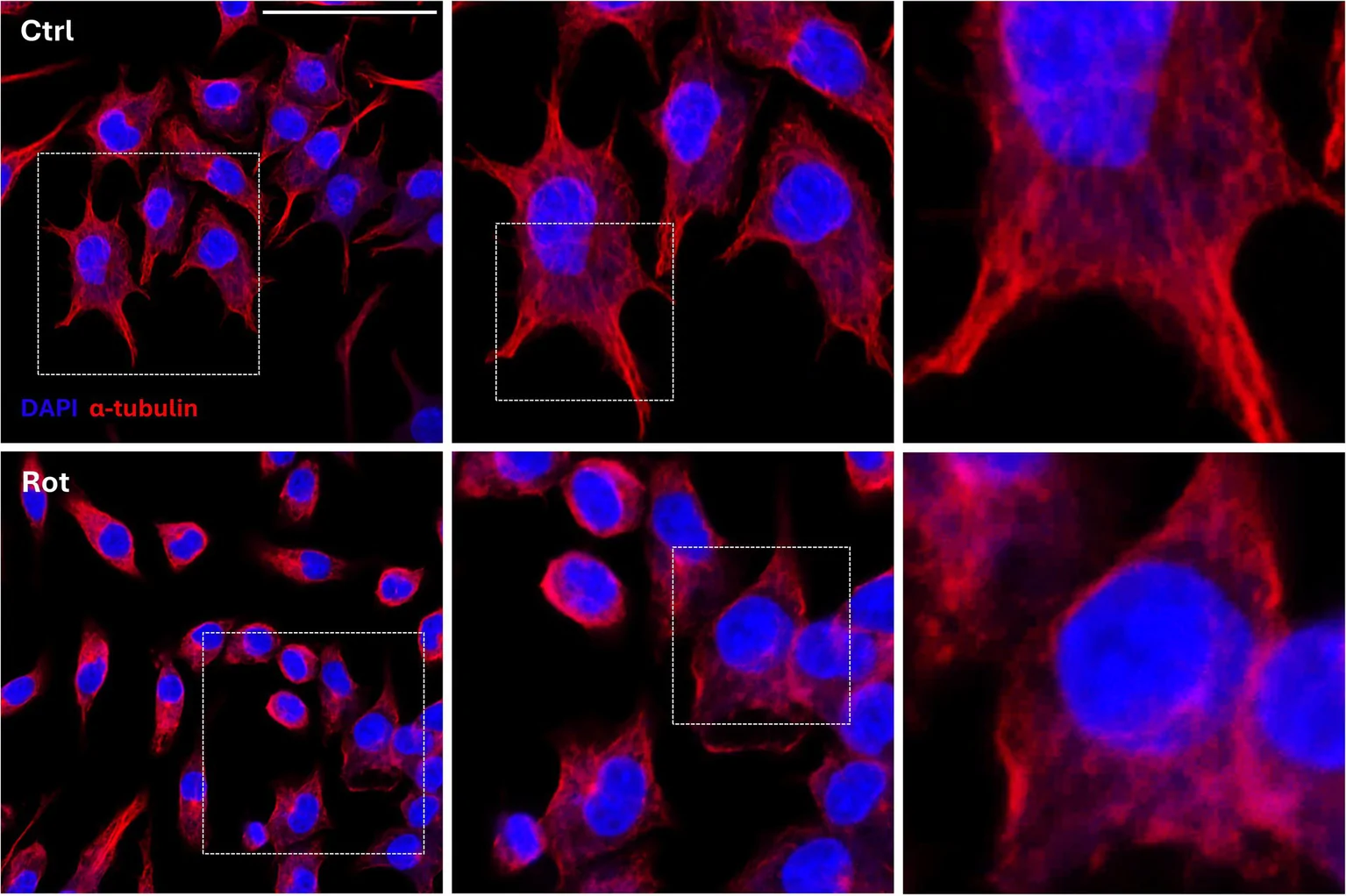
Rotenone affects tubulin organization. Representative fluorescence images of α-tubulin staining in control and 50 nM rotenone-treated BV2 cells, with DAPI nuclear counterstaining. Magnification: 40×. Scale bar = 50 μm.

To further characterize cellular changes, we performed scanning electron microscopy (SEM) was performed (Figure 1C). BV2 cells (Ctrl) displayed a flattened morphology with smooth surfaces and well-developed lamellipodia and filopodia. By contrast, Rot-treated cells showed a more contracted morphology, characterized by reduced spreading, fewer lamellipodia and filopodia, and a roughened cell surface with small irregularities, frequently accompanied by membrane blebbing. These morphological alterations are indicative of cytoskeletal remodeling and cellular stress (Gardiner et al., 2013). Consistent with the SEM findings, immunofluorescence analysis for α-tubulin (Figure 2) revealed a well-organized microtubule network in Ctrl cells, whereas Rot cells showed marked cytoskeletal derangement, with denser tubulin immunoreactivity in the perinuclear region, decreased cellular spreading, and more rounded morphology.

### 3.2 Rotenone-driven mitochondrial network disruption is associated with increased oxidative damage

**Figure 3:**
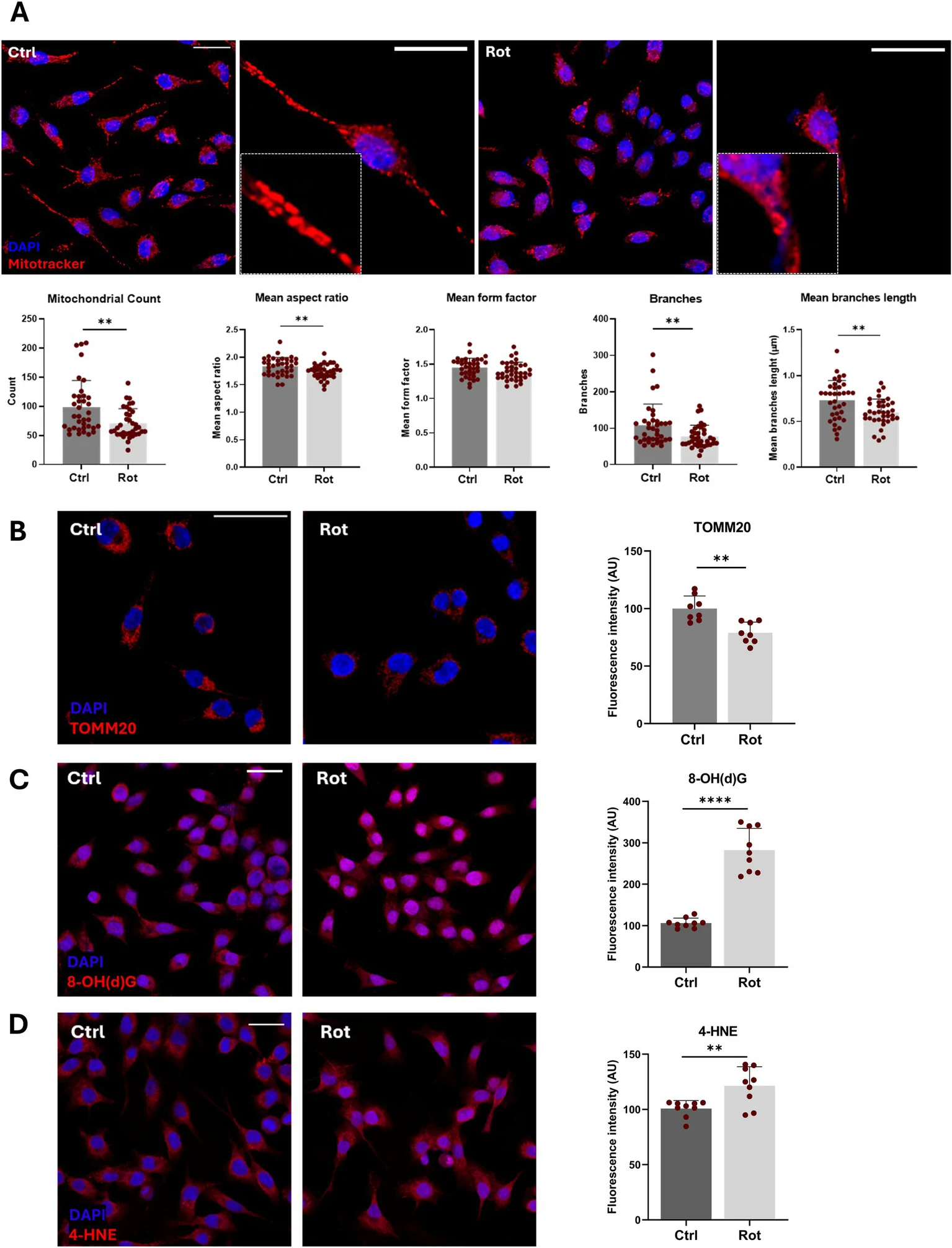
Rotenone induces mitochondrial fragmentation and oxidative stress in BV2 microglial cells. **(A)** Representative confocal microscopy images of BV2 cells treated with vehicle (Ctrl, DMSO) or 50 nM Rot, for 24 h and stained with MitoTracker™ Red CMXRos, to visualize mitochondrial network morphology. DAPI was used for nuclear counterstaining. Quantitative analysis of mitochondrial count, mean aspect ratio, mean form factor, number of branches, and mean branch length is shown below the representative images. A higher-magnification inset of the indicated region is included in the corner to better visualize mitochondrial network details. Dots represent individually analyzed cells from *N* = 9 biological replicates. Magnification: 40×. Scale bar = 25 μm. **(B)** Representative immunofluorescence images of TOMM20-positive BV2 cells, with DAPI nuclear counterstaining, and quantitative analysis of TOMM20 fluorescence intensity. *N* = 8. Magnification: 40×. Scale bar = 50 μm. **(C)** Representative immunofluorescence images of 8-OH(D)G staining, with DAPI nuclear counterstaining, and quantitative analysis of fluorescence intensity. *N* = 9. Magnification: 40×. Scale bar = 25 μm.**(D)** Representative immunofluorescence images of 4-HNE staining, with DAPI nuclear counterstaining, and quantitative analysis of fluorescence intensity. *N* = 9. Magnification: 40×. Scale bar = 25 μm. Data are presented as mean ± SD. Dots represent individual biological replicates. Statistical analysis was performed using an unpaired Student’s *t*-test. ^**^p < 0.01; ^****^p < 0.0001.

Rot treatment induced a profound remodeling of the mitochondrial network, as shown by MitoTracker™ Red CMXRos dye and quantitative morphometric analysis (Figure 3A). Rot-treated cells displayed a significant reduction in mitochondrial count, mean aspect ratio, branch number and mean branch length compared to controls, suggesting a loss of mitochondrial elongation and interconnectivity compatible with mitochondrial fragmentation. This phenotype was further investigated by TOMM20 immunofluorescence (Figure 3B) coupled to quantitative analysis, which confirmed Rot-induced alterations in mitochondrial organization. Given the established role of Rot in promoting mitochondrial dysfunction and ROS generation (Ibarra-Gutiérrez et al., 2023), we next evaluated oxidative damage markers. Rot exposure significantly increased 8-hydroxy-2-deoxyguanosine (8-OH(d)G) immunoreactivity (Figure 3C), indicating enhanced oxidative damage to nucleic acids. In parallel, 4-hydroxynonenal (4-HNE) staining, sometimes concentrated in the perinuclear region, was significantly increased in Rot-treated cells, consistent with enhanced lipid peroxidation (Figure 3D).

### 3.3 Remodeling of the peroxisomal compartment

**Figure 4:**
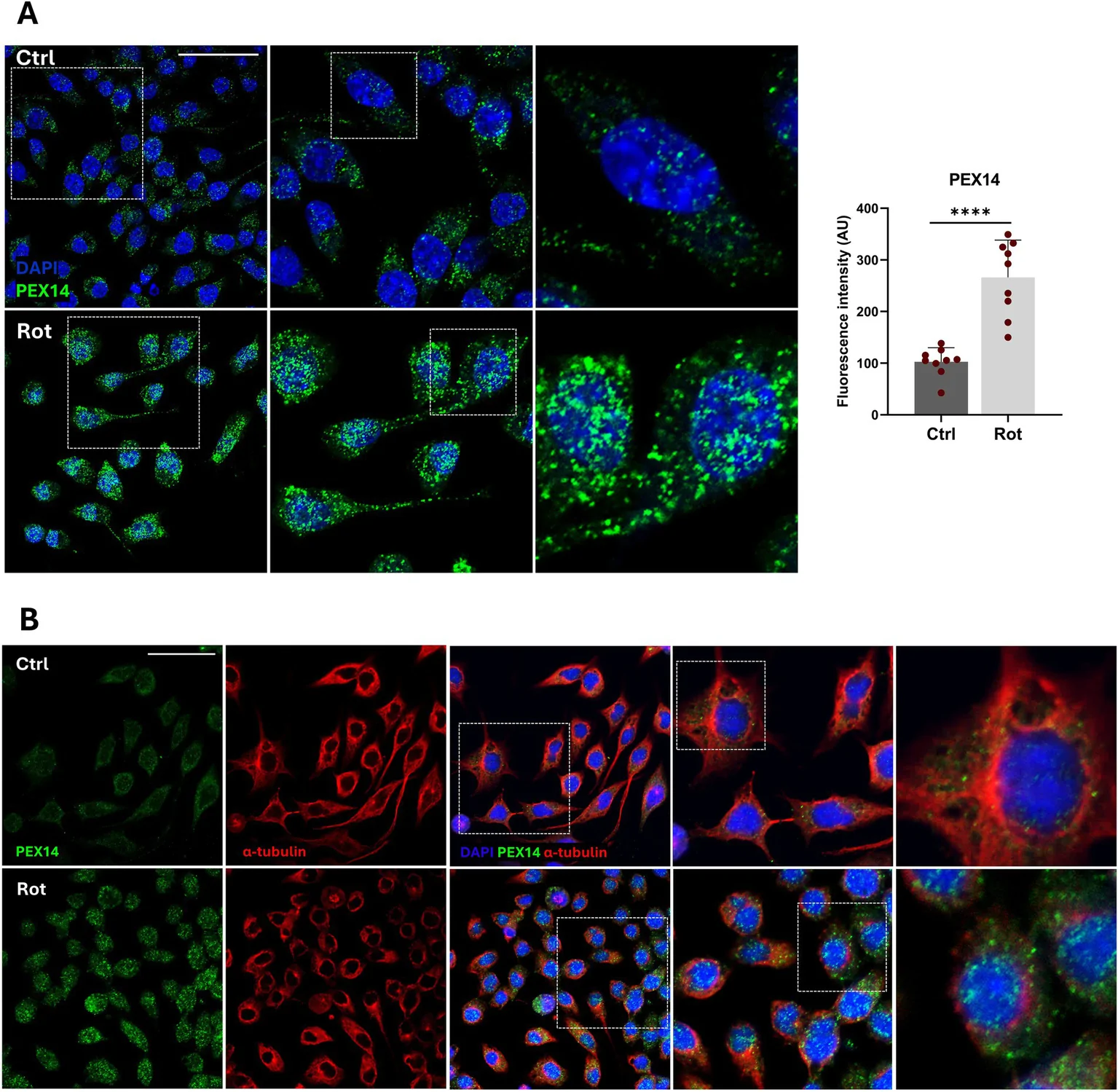
Rotenone induces peroxisomal alterations and affects the intracellular distribution. **(A)** Representative confocal microscopy images of BV2 cells treated with vehicle (Ctrl, DMSO) or 50 nM Rot for 24 h and stained for PEX14. DAPI was used for nuclear counterstaining. A higher-magnification view of the boxed region is shown on the right panel each representative image. Quantitative analysis of PEX14 fluorescence intensity is shown on the right. *N* = 9. Magnification: 40×. Scale bar = 50 μm. **(B)** Representative confocal microscopy images of control and rotenone-treated BV2 cells stained for PEX14 and α-tubulin, with DAPI used for nuclear counterstaining. Single-channel images for PEX14 and α-tubulin are shown together with the merged images. Two sequential higher-magnification views of the indicated regions within the merged images are shown on the right to provide a clearer visualization of PEX14 distribution in relation to the microtubule network. Magnification: 40×. Scale bar = 50 μm. Data are presented as mean ± SD. Statistical analysis was performed using an unpaired Student’s *t*-test. ^****^p < 0.0001.

Considering the well-established crosstalk between mitochondria and peroxisomes in redox homeostasis (Fransen et al., 2017), we next examined whether Rot exposure also affected the peroxisomal compartment. We analyzed the expression and intracellular distribution of Peroxin 14 (Pex14), a membrane protein playing a pivotal role in peroxisomal biogenesis. Immunofluorescence analysis (Figure 4A) revealed a significant increase in Pex14 immunoreactivity, following Rot exposure, suggesting peroxisomal proliferation, under oxidative stress conditions.

To further characterize the effects of Rot in remodeling peroxisomal network, double staining for Pex14 and α-tubulin (Figure 4B) was performed. Confocal microscopy analysis showed redistribution of peroxisomes in Rot-treated cells. Particularly, in control cells, Pex14-positive structures were evenly distributed throughout the cytoplasm along the microtubule network. In contrast, perinuclear accumulation of peroxisomes was detected in response to Rot insult, suggesting altered peroxisomal trafficking.

**Figure 5:**
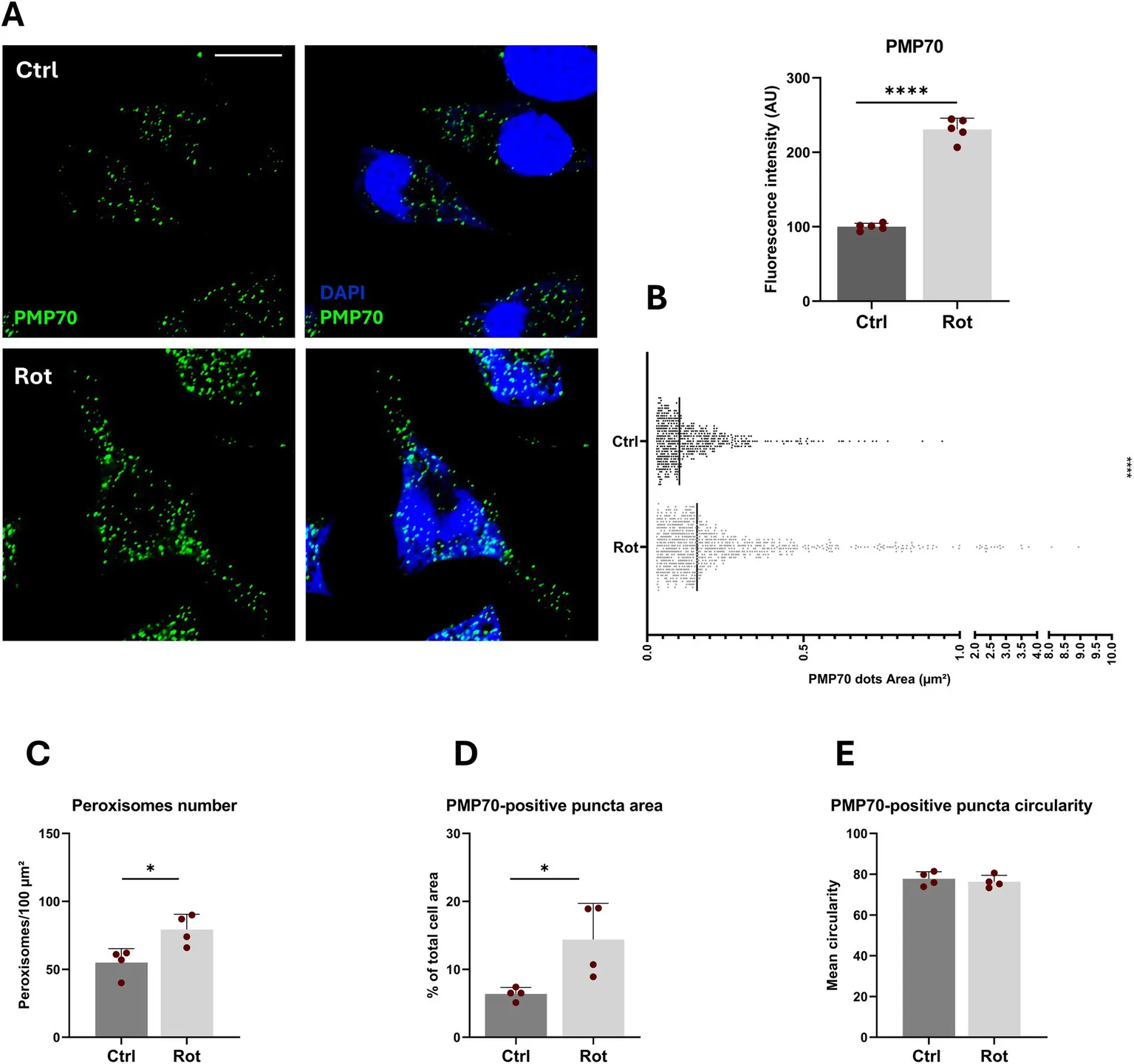
Rotenone promotes remodeling of the PMP70-positive peroxisomal compartment. **(A)** Representative confocal microscopy images of PMP70-positive peroxisomes in control and rotenone-treated BV2 cells, with DAPI nuclear counterstaining. Quantitative analysis of PMP70 fluorescence intensity is shown on the right. *N* = 5. Magnification: 100×. Scale bar = 10 μm. **(B)** Quantitative analysis of PMP70-positive dots area. Dots represent individual PMP70-positive structures analyzed from *N* = 5 biological replicates. Data are presented as mean ± SD. Statistical analysis was performed using an unpaired Student’s *t*-test. ^****^p < 0.0001. **(C)** Quantitative analysis of peroxisome number, expressed as PMP70-positive structures per 100 μm^2^. *N* = 4. **(D)** Quantitative analysis of the total area occupied by PMP70-positive puncta, expressed as a percentage of the total cell area. *N* = 4. **(E)** Quantitative analysis of the mean circularity of PMP70-positive puncta. *N* = 4. Data are presented as mean ± SD, and dots represent individual biological replicates. Statistical analysis was performed using an unpaired Student’s *t*-test. ^*^p < 0.05; ^****^p < 0.0001.

This analysis was subsequently extended to the peroxisomal membrane protein of 70 kDa (PMP70). Immunofluorescence revealed a 2.3-fold-increase in PMP70 signal intensity in Rot-treated cells, as compared to controls (Figure 5A), further suggesting peroxisomal proliferation. Quantitative morphometric analysis showed a significant increase in the average area of PMP70-positive granules (Figure 5B), suggesting enlarged or clustered peroxisomal structures. To further characterize Rot-induced changes in peroxisomal compartment, we performed additional quantitative analyzes of peroxisome number (Figure 5C), total PMP70-positive area relative to cell area (Figure 5D), and PMP70-positive puncta circularity (Figure 5E). Rotenone treatment significantly increased both peroxisome number and the total PMP70-positive area, whereas puncta circularity remained unchanged.

**Figure 6:**
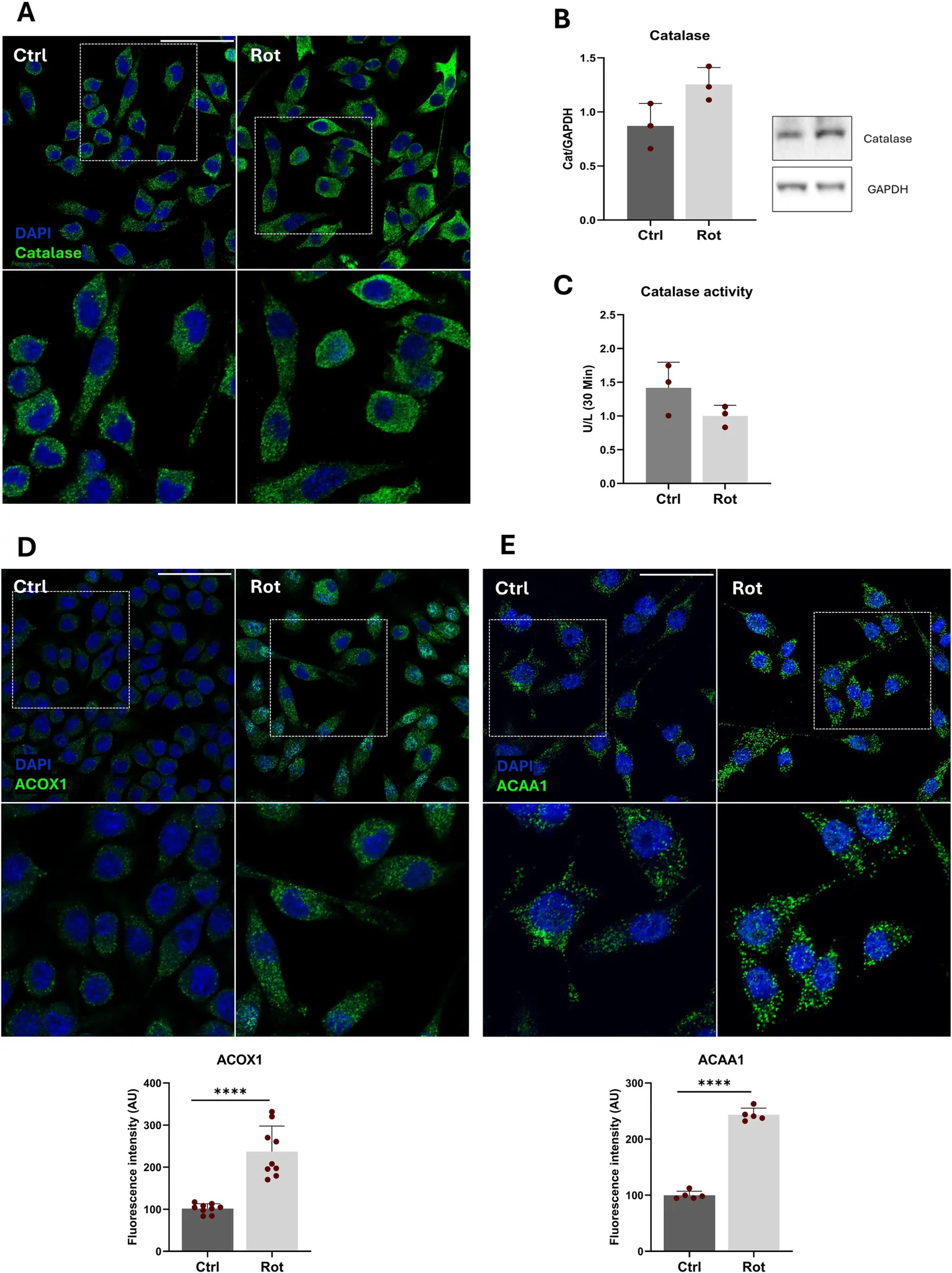
Rotenone effect on catalase and β-oxidation pathways. **(A)** Representative confocal microscopy images of BV2 cells treated with vehicle (Ctrl, DMSO) or 50 nM Rot for 24 h and stained for catalase. DAPI was used for nuclear counterstaining. Higher-magnification views of the boxed regions are shown in the panel below to better visualize the intracellular staining pattern. Magnification: 40×. Scale bar = 50 μm. **(B)** Western blot analysis of catalase in Ctrl and Rot-treated BV2 cells, with GAPDH used as loading control. *N* = 3. **(C)** Quantitative analysis of catalase enzymatic activity in control and rotenone-treated BV2 cells. *N* = 3. **(D)** Representative confocal microscopy images of ACOX1 immunoreactivity in Ctrl and Rot-treated BV2 cells, with DAPI used for nuclear counterstaining. Higher-magnification views of the boxed regions are shown below the corresponding images. Quantitative analysis of ACOX1 fluorescence intensity is shown below. *N* = 9. Magnification: 40×. Scale bar = 50 μm. **(E)** Representative confocal microscopy images of ACAA1immunoreactivity in Ctrl and Rot-treated BV2 cells, with DAPI used for nuclear counterstaining. Higher-magnification views of the boxed regions are shown below the corresponding images. Quantitative analysis of ACAA1 fluorescence intensity is shown below. *N* = 5. Magnification: 40×. Scale bar = 50 μm. Data are presented as mean ± SD. Dots represent individual biological replicates. Statistical analysis was performed using an unpaired Student’s *t*-test. ^****^p < 0.0001.

After detecting alterations in the peroxisomal compartment, we investigated whether Rot exposure even affected peroxisomal metabolism. We first focused on catalase, a major ROS scavenger, involved in hydrogen peroxide detoxification. Both catalase immunofluorescence and Western blot analysis (Figures 6A,B) showed a non-significant trend toward increased catalase levels in Rot-treated cells compared with controls. In immunofluorescence images, catalase displayed a predominantly punctate intracellular pattern, consistent with its peroxisomal localization. However, some cytosolic staining was occasionally detected. Notably, this trend was not accompanied by a corresponding increase in catalase enzymatic activity (Figure 6C). We next investigated peroxisomal fatty acid β-oxidation by examining acyl-CoA oxidase 1 (ACOX1; Figure 6D), the first and rate-limiting enzyme of the pathway, and thiolase acetyl-CoA acyltransferase 1 (ACAA1; Figure 6E), which mediates the final step of the cycle. Immunoreactivity to both markers significantly increased following Rot treatment. Moreover, Rot-treated cells displayed more evident ACAA1-positive structures.

### 3.4 Mitochondria–peroxisome connection

**Figure 7:**
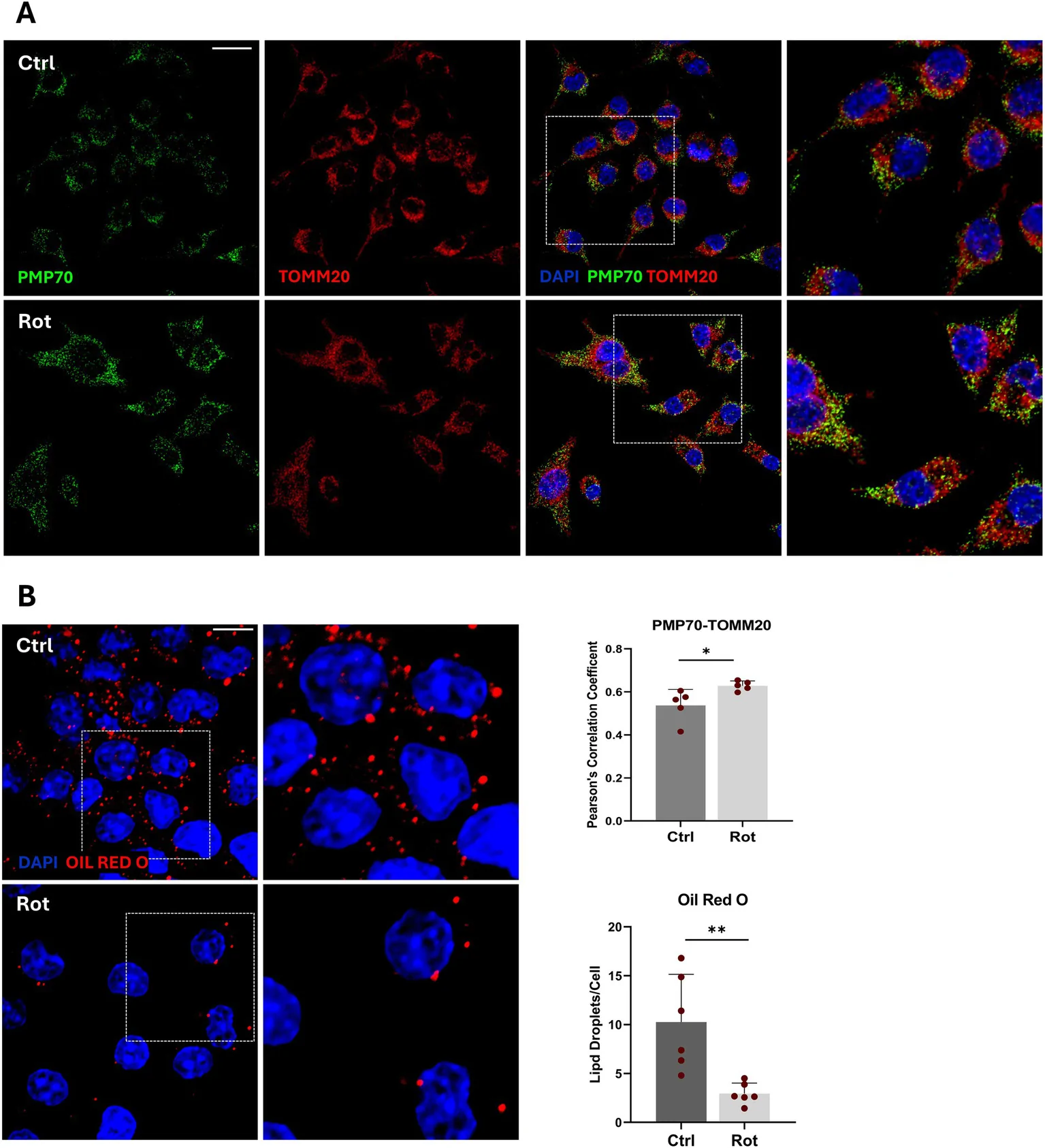
Analysis of mitochondria–peroxisome association and lipid droplets storage. **(A)** Representative confocal microscopy images of BV2 cells treated with vehicle (Ctrl, DMSO) or 50 nM Rot for 24 h and co-stained for PMP70 and TOMM20. DAPI was used for nuclear counterstaining. Single-channel images for PMP70 and TOMM20 are shown together with the merged images. A higher-magnification view of the boxed region is shown on the right panel to better visualize the spatial association between peroxisomes and mitochondria. Quantitative analysis of PMP70–TOMM20 colocalization is reported as Pearson’s correlation coefficient. *N* = 5. Magnification: 40×. Scale bar = 25 μm. **(B)** Representative confocal microscopy images of lipid droplets stained with Oil Red O in Ctrl and Rot-treated cells, with DAPI used for nuclear counterstaining. A higher-magnification view of the boxed region in each image is shown on the right to better visualize individual lipid droplets. Quantitative analysis of the number of lipid droplets per cell is shown on the right. *N* = 6. Magnification: 100×. Scale bar = 10 μm. Data are presented as mean ± SD. Dots represent individual biological replicates. Statistical analysis was performed using an unpaired Student’s *t*-test. ^*^p < 0.05; ^**^p < 0.01.

Double immunofluorescence staining for PMP70 and TOMM20 (Figure 7A) was used to assess the spatial association between peroxisomes and mitochondria. Quantification of signal colocalization showed a significant increase in the Pearson’s Correlation Coefficient in Rot-treated cells compared with controls, indicating an association between the peroxisomal and mitochondrial compartments.

To further study peroxisomal involvement in fatty acid metabolism in our experimental conditions, we examined cellular lipid content using Oil Red O staining. Administration of Rot produced a significant reduction in the number of lipid droplets per cell (Figure 7B), indicating an alteration in lipid droplet homeostasis.

Based on the observed effects of Rot on peroxisomal dynamics, we addressed the involvement of peroxisome proliferator-activated receptor alpha (PPARα), major regulator of peroxisomal biogenesis.

**Figure 8:**
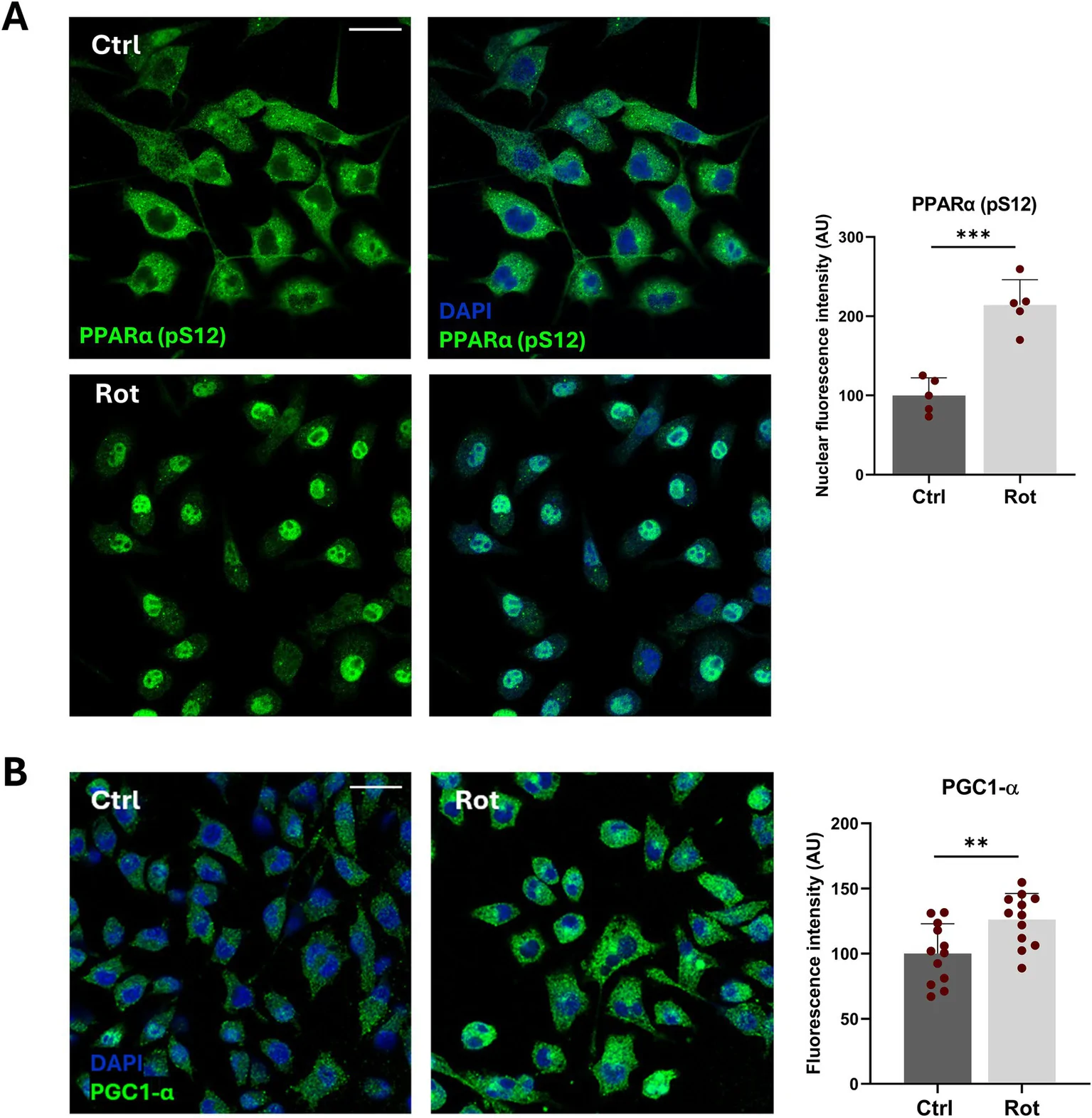
Activation of PPARα signaling. **(A)** Representative confocal microscopy images of BV2 cells treated with vehicle (Ctrl, DMSO) or 50 nM Rot, for 24 h and stained for PPARα (pS12). DAPI was used for nuclear counterstaining. Single-channel images for PPARα are shown together with merged images. Quantitative analysis of nuclear fluorescence intensity is shown on the right. *N* = 5. Magnification: 40×. Scale bar = 25 μm. **(B)** Representative confocal microscopy images of PGC1-α, with DAPI nuclear counterstaining. Quantitative analysis is shown on the right. *N* = 12. Magnification: 40×. Scale bar = 25 μm. Data are presented as mean ± SD. Dots represent individual biological replicates. Statistical analysis was performed using an unpaired Student’s *t*-test. ^**^p < 0.01; ^***^p < 0.001.

Immunofluorescence analysis revealed significantly increased expression of phosphorylated PPARα at Ser12 in Rot-treated cells, compared to controls. Such post-translational modification is associated with transcriptional activation, consistent with its nuclear translocation, following rotenone exposure (Figure 8A). In parallel, PGC1α signal (Figure 8B) was also significantly increased following Rot exposure. Since PGC1α acts as a transcriptional coactivator involved in the regulation of mitochondrial and peroxisomal metabolic programs, these results suggest that Rot treatment promotes activation of a PPARα–PGC1α-related response. Overall, this pathway may contribute to the metabolic adaptation of the peroxisomal compartment in response to Rot-induced mitochondrial dysfunction.

## 4 Discussion

The present study highlights the involvement of peroxisomes in the cellular response to the neurotoxin rotenone (Rot), a natural pesticide and a potent mitochondrial stressor, widely used to reproduce pathological features of PD in *in vivo* and *in vitro* models (Ranasinghe et al., 2024).

We chose BV2 microglial cell line as a suitable model to investigate the cellular changes associated with inflammatory signaling, triggered by Rot-induced oxidative stress. During treatment, both vehicle- and Rot-treated cells were maintained in 2% FBS to reduce serum-derived proliferative and trophic signals. Nevertheless, serum availability may influence the basal BV2 phenotype, as serum deprivation reportedly induce activation-like morphological changes and MAPK signaling (Yao and Fu, 2020), and serum conditions have been shown to modulate redox, mitochondrial, cell-cycle, and pharmacological responses in BV2 cells (Račková and Csekes, 2022). Thus, although identical serum conditions were used for all groups, the contribution of reduced serum to the basal cellular state or to the magnitude of the Rot-induced response cannot be excluded. To establish experimental conditions capable of activating an inflammatory phenotype without causing excessive cytotoxicity, BV2 total cell number was assessed at increasing Rot concentrations. While most tested Rot doses resulted in massive cell death, treatment with 50 nM Rot resulted in a statistically significant, though mild reduction in cell number. Although in contrast with reports on other *in vitro* systems, such susceptibility aligns with recent data obtained in the same cellular model (Li et al., 2024), supporting cell-type dependent sensitivity to Rot. Inhibition of mitochondrial Complex I is known to enhance ROS generation, thereby triggering glial activation (Ferger et al., 2010). Indeed, we observed increased Iba1 expression, a well-established marker of microglial activation (Ito et al., 1998; Jurga et al., 2020). As activated microglia hallmarks PD (Wu et al., 2002), these results further support the suitability of our experimental conditions for modeling inflammatory responses occurring in this disease.

Ultrastructural analysis by SEM confirmed that 50 nM Rot exposure induced dramatic morphological remodeling in BV2 cultures, toward an activated phenotype, consistent with previous evidence from other groups and ours (Dyne et al., 2022; Valenza et al., 2026). While control cells exhibited intact membranes and smooth surfaces with numerous and wide processes, Rot-treated microglia showed pronounced surface alterations, including membrane ruffling and blebbing. Such changes, along with fewer lamellipodia and filopodia, indicate substantial rearrangement of the cytoskeleton, in line with previous studies (Choi et al., 2011; Passmore et al., 2017). Consistent with these findings, immunofluorescence analysis revealed considerable disruption of microtubule organization, characterized by the loss of the typical filamentous tubulin network and marked perinuclear accumulation. Altered microglial cytoskeletal dynamics have been associated with necroptosis, besides disturbed organelle trafficking. Direct action of Rot on cytoskeletal arrangement has long been recognized (Marshall and Himes, 1978). Additionally, Rot-dependent ROS overproduction, consequent to ATP depletion, is known to cause tubulin depolymerization, and to affect cofilin, key component of the actin network (Roy et al., 2023).

Indeed, we demonstrated Rot-induced oxidative damage to macromolecules, as we detected significantly increased levels of 8-OH(d)G and 4-HNE, oxidative modification markers of nucleic acids and lipids, respectively. Consistent with these findings, in our previous study using BV2 cells exposed to the same Rot concentration, MitoSOX™ staining revealed increased mitochondrial superoxide-associated fluorescence (Valenza et al., 2026). Elevated 4-HNE levels specifically reflect the interaction of ROS with polyunsaturated fatty acids (PUFA), leading to the formation of this highly reactive aldehyde as a final product of membrane lipid peroxidation. 4-HNE has been characterized as a double-edged molecule: at low concentrations, it acts as a signaling mediator that promotes cell survival, whereas at higher concentrations, it exhibits cytotoxic effects (Shoeb et al., 2014). The accumulation of lipid peroxidation products suggests deterioration of membrane integrity, which, combined with disruption of redox homeostasis, may contribute to the development of a neurotoxic microenvironment (Barnham et al., 2004). Collectively, these findings support the model of a bidirectional interplay between immune activation and redox imbalance, ultimately amplifying neurodegenerative events.

Rotenone-induced cellular alterations are predominantly associated with mitochondrial damage, which has been thoroughly investigated and documented (Mendes et al., 2023; Currim et al., 2025). Consistently, our MitoTracker™ analysis confirmed that Rot exposure markedly affects mitochondrial architecture, leading to a less elongated and poorly interconnected network, as shown by reduced mitochondrial count, aspect ratio, branching and branch length. In line with these findings, reduced TOMM20 immunoreactivity may reflect the MitoTracker™-detected decrease in mitochondrial abundance, rather than specific downregulation of TOMM20.

Considerably less is known about peroxisomes, despite their fundamental role in redox balance and lipid metabolism and their close interaction with mitochondria (referred to as the “peroxisome–mitochondria connection”) (Fransen et al., 2017; Islinger et al., 2018). At the gene regulation level, both organelles are jointly governed by cis- and trans-acting factors, including PPARα and its coactivator PGC-1α, which serve as major regulators of their biogenesis and function (Rius-Pérez et al., 2020). Furthermore, mitochondria and peroxisomes share several molecular components involved in organelle dynamics, particularly fission and, to a lesser extent, fusion (Castro et al., 2018; Schrader, 2006).

A novel finding of our study is that Rot exposure results in peroxisomal proliferation, as assessed by membrane markers Pex14 and PMP70, dependable indicators of peroxisomal population (Grant et al., 2013). Indeed, peroxisomal count resulted higher following Rot treatment and immunoreactive puncta showed enhanced concentration and extended on a wider area of the cell. Increased peroxisomal abundance was accompanied by activation of the PPARα signaling pathway, as demonstrated by its upregulation and nuclear translocation, underscoring its function as a major regulator of peroxisomal biogenesis and metabolism. Such pathway, strongly activated in rodents, is compatible with the mouse origin of BV2 cells, while extending this interpretation to human cells should be cautious, in view of the limited effects of PPARα activation on peroxisomes in primates (Lawrence et al., 2001).

From a pathophysiological perspective, the observed increase in peroxisome mass may represent an adaptive response to Rot-induced mitochondrial damage, as a compensatory mechanism to enhance antioxidant defenses. Previous studies have shown that increased peroxisome numbers correlate with greater resistance to ROS-induced damage, whereas reduced proliferation exacerbates cellular vulnerability (Delmaghani et al., 2015). In microglia, maintaining intracellular redox balance is essential to prevent excessive cytotoxicity, as these cells are sensitive to oxidative and inflammatory *stimuli* and are themselves sources of reactive oxygen and nitrogen species (Qin et al., 2023).

In this context, possible contribution of peroxisomes to microglial redox homeostasis could be suggested by the observed activation of PPARα, reportedly promoting the expression of antioxidant enzymes (Shin et al., 2016). However, in our experimental conditions, catalase showed only a non-significant increasing trend by immunofluorescence and Western blot, while its enzymatic activity remained unchanged, indicating that this peroxisomal response may not be sufficient to fully counteract redox imbalance. The reason for a functionally limited catalase involvement, possibly related to redox-dependent or post-translational mechanisms triggered by oxidative stress, remains to be ascertained (Baker et al., 2023). Regardless, PPARα-mediated induction of other cellular ROS scavenging pathways can be hypothesized as well.

Peroxisomes exhibit a remarkable capacity to respond to environmental and cellular cues, enabling rapid modulation of their abundance, morphology, and functions (Carmichael and Schrader, 2022). Besides changes in size and number, peroxisomes undergo significant shifts in intracellular localization, including long-range movement that depends on microtubules (Schrader et al., 1996). Under normal conditions, in BV2 cells, peroxisomes are typically distributed throughout the cytoplasm; however, exposure to Rot results in a pronounced perinuclear accumulation. Double immunofluorescence analyzes of Pex14 and tubulin demonstrated that such redistribution reflects the above-described cytoskeletal alterations, causing disrupted intracellular trafficking. Comparable alterations in peroxisomal trafficking have been reported in conditions such as AD, in which microtubule disruption contributes to peroxisomal dysfunction (Kou et al., 2011). Our findings are also consistent with those by Passmore et al. (2017) on monkey kidney cells, showing that Rot treatment induces peroxisomal clustering, concurrent with microtubule disorganization. Even in our model, Rot-treated cells exhibited significantly increased size of PMP70-positive *puncta*, indicating potential organelle clustering. Peroxisome biogenesis can be induced by various *stimuli*, including growth factors, fatty acids, and ROS, conditions that promote dynamic remodeling of the organelles in response to changing cellular metabolic demands (Schrader and Fahimi, 2006; Schrader et al., 2016).

Given the high concentration of lipids in the brain, peroxisomes are central to neural cells lipid turnover. Specifically, they operate the β-oxidation of various fatty acid substrates, including those with very long or branched carbon chains, and generate precursors necessary for PUFA biosynthesis. Additionally, peroxisomes facilitate essential reactions in the production of ether phospholipids, or plasmalogens, which are fundamental components of neuronal and myelin structures (Kumar et al., 2024). Consistent with their contribution to the processing and turnover of membrane-associated lipids, peroxisomes are typically located next to neuronal plasma membrane (Wang et al., 2018). Notably, astroglial cells display different patterns of peroxisome distribution (Rhoads et al., 2025), while no study so far addressed the intracellular localization of these organelles in microglia. Confocal microscopy analysis of enzymatic markers associated with fatty acyl β-oxidation demonstrated an induction of this catabolic pathway, primarily governed by the rate-limiting enzyme ACOX1 and ACAA1 (Tahri-Joutey et al., 2021). These results indicate that peroxisomes may compensate for mitochondrial dysfunction, which proper lipid metabolism is impaired. Noteworthy, peroxisomes have been demonstrated to be capable of metabolizing medium and long-chain fatty acids (Violante et al., 2019), generally oxidized by mitochondrial pathway. However, peroxisomal β-oxidation does not generate ATP but produces heat, thereby limiting the metabolic support provided by peroxisomes (Liu et al., 2025).

In line with this interpretation, the increased Pearson correlation coefficient between PMP70- and TOMM20-positive compartments observed after Rot exposure suggests a closer mitochondrion–peroxisome interplay. This finding is consistent with previous studies describing mitochondria and peroxisomes as functionally and physically connected organelles (Fransen et al., 2017; Shai et al., 2018). In this context, the organelle proximity observed in Rot-treated cells may facilitate metabolic exchange between the two compartments, potentially supporting both fatty acid handling and redox homeostasis. Indeed, Kim’s group recently demonstrated that peroxisome–mitochondria contacts increase during mitochondrial oxidative stress and contribute to mitochondrial redox homeostasis by enabling the transfer of mitochondrial ROS to the peroxisomal lumen (DiGiovanni et al., 2025). It will be interesting to further investigate the proximity and interaction of these organelles at a higher resolution—e.g., at the ultrastructural level—to confirm altered peroxisome-mitochondria contacts.

Our data on Oil Red O staining, which selectively labels neutral lipids, demonstrated a significant reduction in lipid droplet content in BV2 microglial cells following Rot treatment. This observation is plausibly related to the increased expression of β-oxidation enzymes, and consistent with previous studies, reporting a reduction in Oil Red O–positive area in cells treated with 100 nM Rot (Zhang et al., 2013). Thus, Rot insult seems to promote lipid mobilization, indicating a distinct metabolic response to the mitochondrial deficit, that can rapidly elevate cellular energy demand. Under such acute stress, very long chain fatty acids stored as triglycerides in lipid droplets can be mobilized and rapidly transported to peroxisomes for initiation of fatty acid oxidation, then utilized in pathways for energy production. Interactions between lipid droplets and peroxisomes may contribute to the coordination of lipid trafficking and fatty acid metabolism; however, whether the observed lipid droplets decrease reflect enhanced peroxisomal β-oxidation, reduced lipid droplet biogenesis, or increased lipid droplet degradation, remains to be determined. It should be however mentioned that chronic oxidative and inflammatory environments characterizing neurodegenerative conditions, have been instead associated to increased lipid droplet accumulation, which has been proposed to serve protective roles by sequestering excessive lipids, limiting lipid peroxidation, and functioning as long-term lipid reservoirs (Valadas et al., 2018; Yin, 2023).

Overall, the results of our study underscore the remarkable plasticity of the peroxisomal compartment, suggesting its capacity to cope with mitochondrial damage, through morpho-functional changes affecting ROS metabolism and lipid homeostasis. Particularly, the data presented here further highlight the bidirectional relationship linking these two classes of organelles. It is also worth mentioning that peroxisomal lipid metabolism is not limited to energy management but impacts more broadly neurodegeneration-related events. Indeed, crucial signaling molecules such as prostaglandins, leukotrienes and tromboxanes, are lipid substrates for β-oxidation pathway, suggesting peroxisomal entanglement in neuroinflammation. Further studies are needed to clarify whether targeting peroxisomal pathways could serve as a potential strategy to mitigate the energetic dysmetabolism and neuroinflammatory processes associated with PD and other neurodegenerative disorders.

## Data Availability

The raw data supporting the conclusions of this article will be made available by the authors, without undue reservation.
